# Grading of Castleman Disease Histopathology with an Attention-Based Multiple Instance Learning Model

**DOI:** 10.3390/diagnostics16172750

**Published:** 2026-08-27

**Authors:** Muir J. Morrison, Alnoor, Brendan O’Fallon, Ashley Hutchings, Mark Dewey, Paul English, Alexandra E. Rangel, Lauren M. Zuromski, Katie Knight, Anna Bowen, Kiera Kearns, Kristin Shaw, Janani Sankar, Oscar Silva, Peyman Z. Samghabadi, Olga K. Weinberg, Miguel D. Cantu, Yidan Xu-Monette, Archana Agarwal, Timothy M. Hanley, Kristin H. Karner, Madhu Menon, Rodney R. Miles, Jay L. Patel, Anna Shestakova, Peng Li, Nicholas C. Spies, Ken H. Young, David P. Ng, Robert S. Ohgami

**Affiliations:** 1 ARUP Laboratories, Institute for Research and Innovation in Diagnostic and Precision Medicine™, Salt Lake City, UT 84108, USA; 2 Department of Pathology, University of Miami Miller School of Medicine, Miami, FL 33136, USA; 3 Department of Pathology, Stanford University, Stanford, CA 94305, USA; 4 Department of Pathology, University of California, San Francisco, San Francisco, CA 94110, USA; 5 Department of Pathology, University of Texas Southwestern Medical Center, Dallas, TX 75235, USA; 6 Department of Pathology, Duke University School of Medicine, Durham, NC 27710, USA; 7 Department of Pathology, University of Utah, Salt Lake City, UT 84112, USA

**Keywords:** Castleman disease, idiopathic multicentric Castleman disease, digital pathology, whole slide imaging, multiple instance learning, pathology foundation models, ordinal grading

## Abstract

**Background/Objectives**: Castleman disease is a rare cytokine-driven lymphoproliferative disorder in which lymph node histopathology provides key diagnostic information. Morphologic features are graded semiquantitatively and often show substantial interobserver variability. **Methods**: We developed an automated approach to grade six Castleman disease-associated histologic features on hematoxylin and eosin (H&E) whole slide images (WSIs) using an attention-based multiple instance learning (MIL) model built on a large pathology foundation model encoder. A multi-institution cohort comprised 544 lymph node WSIs, including 397 from cases with Castleman disease or Castleman-like histology and 147 from cases without suspicion for Castleman disease. These case ascertainment categories were not model prediction targets. Slides were assigned ordinal grades (0–3) for regressed germinal centers, follicular dendritic cell prominence, increased vascularity, hyperplastic germinal centers, plasmacytosis, and follicular twinning by hematopathologists. Model performance was assessed on a held-out evaluation set of 142 WSIs. **Results**: Across the six features, accuracy ranged from 0.52 to 0.68 (mean 0.60). Disagreements were predominantly minor: 96% of predictions were within one grade of the reference. Feature-specific tile contribution heatmaps showed qualitative spatial correspondence with selected plasma cell-rich, vessel-rich, and follicular regions but were not evaluated as quantitative feature localization maps or causal explanations. In a preliminary reader study, concordance with the reference varied widely among hematopathologists (Krippendorff’s alpha 0.20–0.95, mean 0.50), with the model demonstrating concordance in the mid-range (0.56). **Conclusions**: These findings support the feasibility of automated grading of Castleman disease-associated histologic features for research standardization. The model does not classify Castleman disease, and its potential use as an input to diagnostic or clinical trial workflows requires evaluation in separately designed studies with adjudicated diagnostic labels and integrated clinical data.

## 1. Introduction

Castleman disease (CD) encompasses a group of uncommon cytokine-driven lymphoproliferative disorders defined by characteristic lymph node morphologic changes and a spectrum of clinical sequelae. Population-based estimates suggest an annual incidence on the order of tens of cases per million person-years for unicentric CD and single-digit cases per million person-years for idiopathic multicentric CD (iMCD) [1,2].

Across CD subtypes, lymph node histopathology is critical: it is currently required for a diagnosis of iMCD and provides essential information to distinguish CD from mimics such as autoimmune and infectious lymphadenopathies, IgG4-related disease, and lymphomas. Indeed, consensus criteria for iMCD explicitly require compatible lymph node histopathology, with five commonly used morphologic features that are scored semi-quantitatively: regressed germinal centers, follicular dendritic cell (FDC) prominence, increased vascularity, hyperplastic germinal centers, and plasmacytosis. An additional feature, follicular twinning, has been observed in lymph nodes involved in CD, although it is not currently incorporated into formal histologic grading frameworks. For iMCD, compatible lymph node histopathology is one of two required major diagnostic criteria. The histopathologic major criterion requires grade 2 or 3 regressed germinal centers or plasmacytosis, together with other compatible findings within the iMCD histologic spectrum. The diagnosis also requires multicentric lymphadenopathy, at least two minor clinical or laboratory criteria including at least one laboratory abnormality, and exclusion of relevant mimics [3]. Thus, these histologic feature grades inform, but do not independently establish an iMCD diagnosis. Because these features frequently co-occur and vary within lymph nodes, their evaluation relies on subjective thresholding (even among experienced observers) and semi-quantitative scoring systems, which, consequently, show substantial interobserver variability, limiting their reliability as reproducible endpoints for clinical research and disease registries [3,4]. 

Clinically, CD is subdivided into unicentric disease, in which symptoms are driven primarily by a single enlarged lymph node region, and multicentric disease, characterized by diffuse lymphadenopathy with systemic inflammatory manifestations [5,6,7]. Multicentric disease is further classified into human herpes virus 8 (HHV-8)-associated, POEMS (polyneuropathy, organomegaly, endocrinopathy/edema, monoclonal protein, and skin changes)-associated, and idiopathic. Although these clinical syndromes differ, lymph node histopathology is central to all subtypes and commonly spans a continuum between so-called hyaline vascular and plasmacytic patterns, with mixed and intermediate forms frequently encountered. Because these patterns form a continuum rather than discrete categories, categorical diagnosis is challenging, and assessment of individual histologic features provides a more faithful representation of what is observed at the microscope [3,4]. 

For iMCD specifically, an accurate diagnosis has direct therapeutic consequences. IL-6-directed therapy is the recommended first-line treatment for iMCD in appropriate clinical contexts, and a randomized, placebo-controlled trial of siltuximab demonstrated durable tumor and symptomatic responses in 34% of treated patients compared with 0% in the placebo arm [8,9]. Despite consensus guidelines, real-world claims data indicate underutilization of IL-6-directed therapy, consistent with ongoing barriers to case identification and subtype confirmation [2,10]. Improving the reliability of histopathologic assessment is therefore not a purely academic exercise; it is linked to timely access to disease-modifying therapy and to trial eligibility [10,11,12,13]. 

Digital pathology and weakly supervised learning provide a route to objective, scalable morphologic quantification. Multiple instance learning (MIL) is well suited to whole slide images (WSIs), where slide-level labels can be learned without dense region annotations by aggregating information across many tissue tiles. Attention-based MIL methods not only improve performance but also produce attention maps that can be interrogated for interpretability, aligning naturally with the needs of hematopathology [14,15,16,17,18,19]. Recent work has shown that data efficiency improves substantially when tile representations are derived from large, self-supervised foundation models trained on diverse histopathology data, including both vision-only and multimodal vision-language models [18,19,20,21]. Crucially, these pathology foundation models can be used as “off-the-shelf” feature extractors without fine-tuning, which is ideal for our data-limited setting due to the rarity of CD. However, most published computational pathology systems focus on tumor detection or tumor subtyping; there are comparatively few examples targeting rare, non-neoplastic lymph node disorders, where datasets are smaller and where the outputs are ordinal histologic grades rather than binary diagnoses [14,19,20,22,23,24,25]. 

From an informatics standpoint, CD is a useful stress test for computational pathology. The disease is rare, the morphologic signal is spatially heterogeneous, and the relevant targets are often present in variable proportions across the slide rather than confined to a single lesion focus. These characteristics are poorly matched to conventional supervised pipelines that rely on region annotations or on uniform tissue distributions. An attention-based MIL framework can directly address this mismatch by allowing the algorithm to learn which slide regions drive each feature grade, while still producing a single, reproducible output per case [14,22].

In this study, we report an attention-based MIL model that grades six CD-associated histologic features on hematoxylin and eosin (H&E)-stained WSIs. The model was designed to quantify these morphologic features and was not trained to classify Castleman disease versus non-Castleman disease. The model is built on embeddings from Virchow2, a mixed-magnification pathology foundation model trained on more than three million WSIs [19]. We evaluate model performance against reference grades and compare its agreement to that of multiple hematopathologists. We also present feature-specific tile contribution heatmaps as qualitative visualizations of model weighting [14,17]. 

## 2. Materials and Methods

### 2.1. Study Cohort and Whole Slide Images

Archival H&E-stained lymph node sections were retrospectively identified from ARUP Laboratories, Duke University, the University of Texas Southwestern Medical Center, the University of California, San Francisco, and Stanford University between 1990 and 2025. The cohort comprised 544 WSIs, including 397 from cases with Castleman disease (CD) or CD-like histology and 147 from cases in which the original pathology diagnosis and diagnostic comment did not identify or suggest CD and for which there was no clinical suspicion for CD. These categories describe how cases were ascertained and were not used as diagnostic prediction targets. The comparison cases were not systematically adjudicated as diagnostic negative controls. Cases were drawn from excisional biopsies and resections performed as part of routine clinical care. Core biopsies were excluded when tissue area was insufficient for reliable assessment of follicular architecture. Tissue processing and H&E staining followed the routine clinical protocols of the contributing institutions and were not prospectively harmonized; detailed historical staining parameters were unavailable for some archival cases. All slides were subsequently digitized using the same physical Pramana HT4 scanner at 0.25 µm/pixel (40× optical magnification), de-identified before analysis, and used under institutional review board approval with a waiver of consent. 

### 2.2. Histologic Grading and Reference Standard

Each WSI was graded on an ordinal 0–3 scale for six morphologic features commonly used in the evaluation of CD: regressed germinal centers, follicular dendritic cell (FDC) prominence, increased vascularity, hyperplastic germinal centers, plasmacytosis, and follicular twinning. Grading followed WHO-HAEM5 and international consensus criteria for CD, where applicable [3,5,6,26,27]. Grade 0 corresponded to absent or within normal limits, grade 1 to mild, grade 2 to moderate, and grade 3 to marked or diffuse involvement. Two reference hematopathologists with focused experience in CD histopathology and application of the grading framework (F.A. and R.S.O.) initially graded slides separately using the same six-feature rubric. Because the features may be spatially heterogeneous, grades reflected the predominant overall pattern and extent across the entire tissue section rather than the highest-grade finding in an isolated focus. Cases with substantive discrepancies or recognized interpretive uncertainty were jointly reviewed and adjudicated by consensus. The resulting grades served as the consensus reference for model evaluation. The reference graders did not participate in the independent reader study.

### 2.3. Dataset Partitioning and Reader Study

To prevent information leakage, training/validation partitioning was performed at the case level to ensure multiple WSIs from the same case were placed in the same split. For ARUP data, train/validation splits were stratified by the reference grades of their CD-like histology. Non-ARUP data, for which case information was unavailable, were grouped by institution and placed entirely in the training or validation split, again to ensure no train/validation data leakage. The final training and validation sets had 402 and 142 WSIs, respectively. To contextualize model performance relative to human variability, a subset of cases was independently graded by seven rater hematopathologists in addition to the reference graders. All raters applied the same six-feature, 0–3 ordinal rubric and were blinded to clinical information, reference grades, and model outputs. Inter-rater agreement was quantified using Krippendorff’s alpha [28]. 

### 2.4. Image Preprocessing, Embeddings, and Slide-Level Modeling

**Figure 1:**
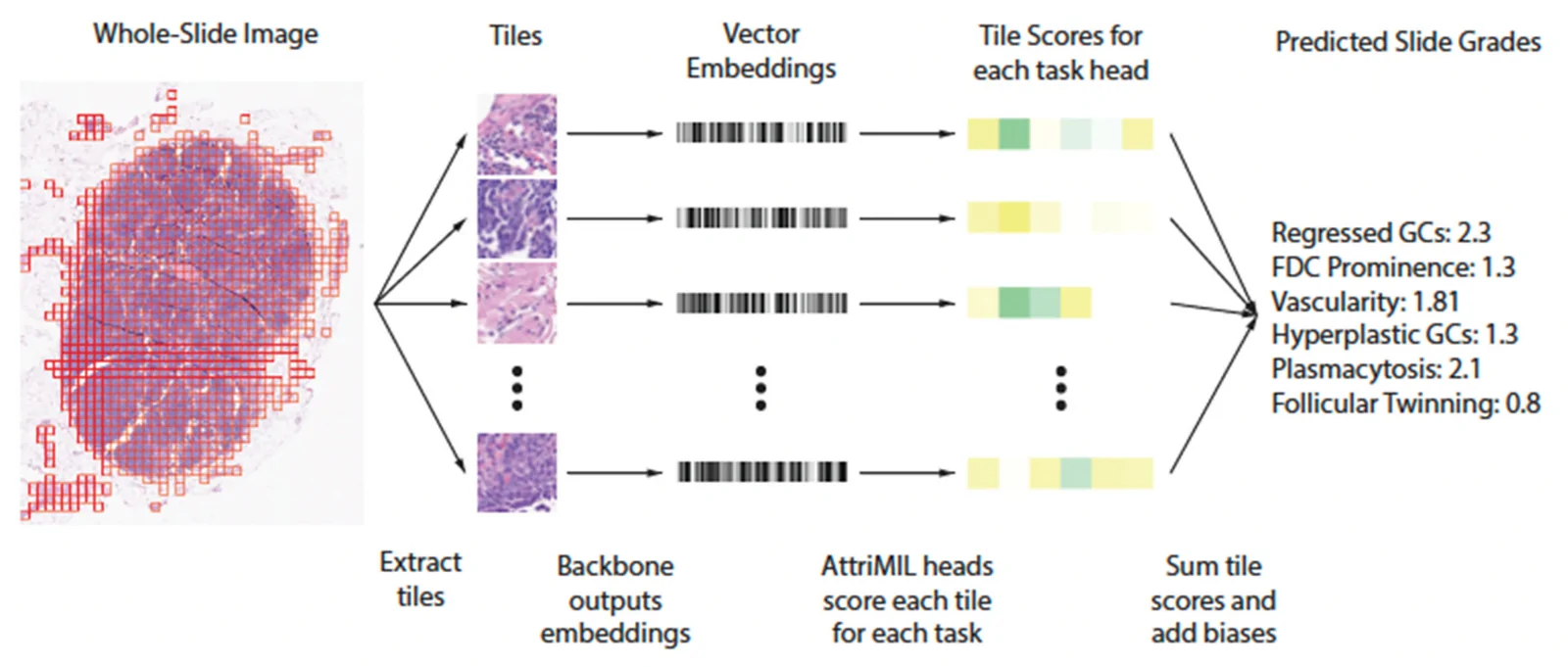
Overview of the computational pipeline for slide-level histologic feature grading. H&E whole slide images are segmented to identify tissue and tiled into patches. Tiles are encoded with Virchow2, and an attention-based MIL model aggregates tile embeddings to predict ordinal grades (0–3) for six Castleman disease-associated histologic features.

WSIs were processed using a standard weakly supervised pipeline. Tissue regions were identified at low magnification and tiled into fixed-size image patches, with background and technical artifacts removed using simple HSV color space filters. Each retained tile was encoded as a feature vector using Virchow2, a self-supervised pathology foundation model trained on more than three million WSIs across multiple magnifications [19]. Virchow2 was selected as a pragmatic frozen encoder because its large mixed-magnification pretraining corpus and compatibility with the 0.5 µm/pixel tile resolution were well suited to the multiscale architecture of lymph nodes. This selection was not based on a head-to-head comparison with other encoders. Slide-level feature grades were predicted using attention-based multiple instance learning (MIL) [14,17]. The six ordinal feature grades were the only model targets; no CD versus non-CD classifier was trained. Each slide was represented as a bag of tile embeddings, and a multi-task regression model generated six continuous feature scores, one per histologic feature. Regression was selected because the grades are ordered and the magnitude of disagreement is relevant; an adjacent grade discrepancy is smaller than a difference in two or three grades. Continuous outputs ranged from −0.5 to 3.5 and were rounded to the nearest integer only for comparison with the discrete 0–3 expert-consensus reference grades. Ordinal specific objectives were not compared in the present study. Figure 1 provides an overview of the computational workflow from a raw WSI to slide-level predictions.

### 2.5. Model Interpretability and Performance Evaluation

To visualize model weighting, feature-specific signed tile contribution scores were mapped back to their spatial coordinates on the WSI and displayed as normalized overlays. Positive and negative scores indicate relative contributions toward higher and lower continuous feature scores, respectively. Values were normalized within each feature-specific map and should not be compared quantitatively across panels. The overlays were used for qualitative inspection and were not treated as causal explanations, validated feature localization maps, or histologic segmentations.

Model performance was assessed on the held-out evaluation set of 142 WSIs with complete reference grades across all six histologic features. Ordinal confusion matrices were used to summarize exact agreement and the magnitude of disagreement between predicted and reference grades. Agreement between model predictions and reference grades, as well as interobserver agreement among human raters, was quantified using Krippendorff’s alpha [28]. 

## 3. Results

### 3.1. Six Ordinal Grades in a Fully Annotated Held-Out Evaluation Set

Virchow2 tile embeddings were aggregated by an attention-based multiple-instance learning model to generate six ordinal grades per WSI. Performance was assessed on a held-out evaluation set of 142 WSIs with complete reference grades across all six histologic features, yielding a total of 852 feature-level predictions.

### 3.2. Reference Grading Shows Feature Imbalance and Human Variability

Reference grade distributions differed substantially by feature (Table 1). Hyperplastic germinal centers and follicular twinning were most frequently graded as absent or minimal, with grade 0 assigned in 84/142 (59.2%) and 86/142 (60.6%) cases, respectively. In contrast, regressed germinal centers were more often severe, with grade 3 assigned in 60/142 (42.3%) cases. Because of these imbalances, exact accuracy alone can be misleading, and performance is better assessed by examining the size of grading disagreements rather than simple right or wrong classification.

In the reader study, pairwise agreement with the reference varied widely among hematopathologists, with Krippendorff’s alpha ranging from 0.203 to 0.952 (Table 2). The model’s agreement with the reference (alpha 0.560) fell within the range observed among the participating readers. This comparison provides context for model reference agreement but should not be interpreted as equivalence between the model and a practicing hematopathologist.

### 3.3. Model Demonstrates Strong Ordinal Agreement with Predominantly “Off-by-One” Errors

**Figure 2:**
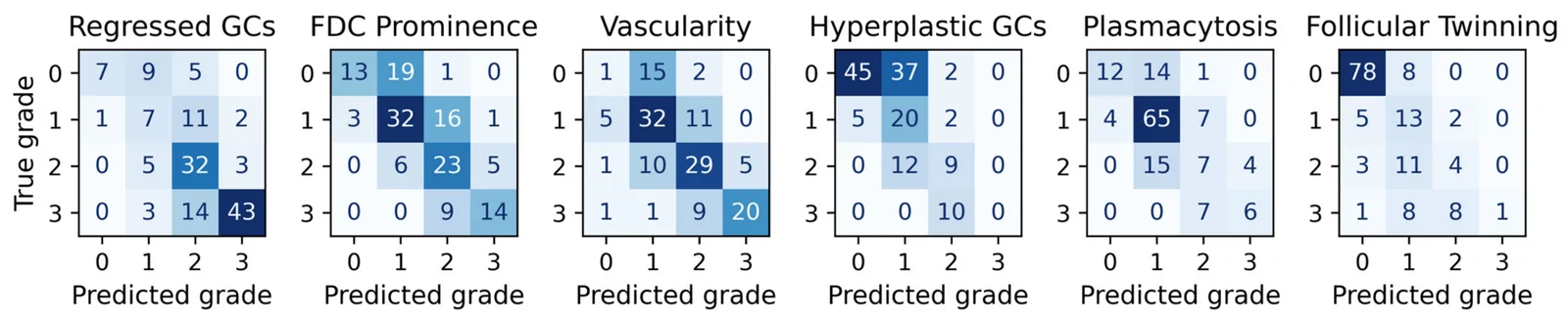
Confusion matrices for model predictions versus reference grades on the held-out evaluation set (*n* = 142 WSIs). Rows indicate the reference grade and columns indicate the model-predicted grade for each of six histologic features. Color-coding is proportional to the number of samples per bin.

Confusion matrices for each histologic feature are shown in Figure 2. Across all six features, disagreements between model-predicted and reference grades were predominantly limited to adjacent ordinal categories, consistent with the graded nature of the task. Visual inspection of the confusion matrices demonstrated that misclassifications were rarely large and instead reflected one-grade shifts in most cases.

Across all features, 96.2% of predictions were within one grade of the reference, and only 32 of 852 predictions (3.8%) differed by two or more grades. Large discrepancies were uncommon for plasmacytosis (1/142 predictions) and follicular dendritic cell prominence (2/142) and were most frequent for follicular twinning (12/142) and regressed germinal centers (10/142), where the full 0–3 grading range was represented among reference grades (Table 1).

### 3.4. Feature-Specific Errors Reveal Predictable and Interpretable Grading Patterns

Grade-specific analyses revealed systematic tendencies that were not apparent from aggregate agreement summaries. For vascularity, cases graded as 0 by the reference graders were rarely predicted as grade 0 by the model (1 of 18 cases), with most shifted to grade 1 (15 of 18 cases), indicating a tendency toward mild overcalling at the low end of the scale. In contrast, follicular twinning showed strong recognition of grade 0 (78 of 86 cases) but poor identification of grade 3, with only 1 of 18 reference grade 3 cases predicted as grade 3. Hyperplastic germinal centers demonstrated a similar compression toward intermediate grades: none of the ten reference grade 3 cases were predicted as grade 3, and many reference grade 0 cases were shifted upward to grade 1 (Figure 2). These feature-specific patterns indicate that model errors were structured rather than random and tended to reflect conservative shifts toward adjacent grades, particularly for features with imbalanced reference distributions or limited representation of extreme grades.

### 3.5. Tile Contribution Overlays Show Qualitative Spatial Correspondence with Histologic Morphology

**Figure 3:**
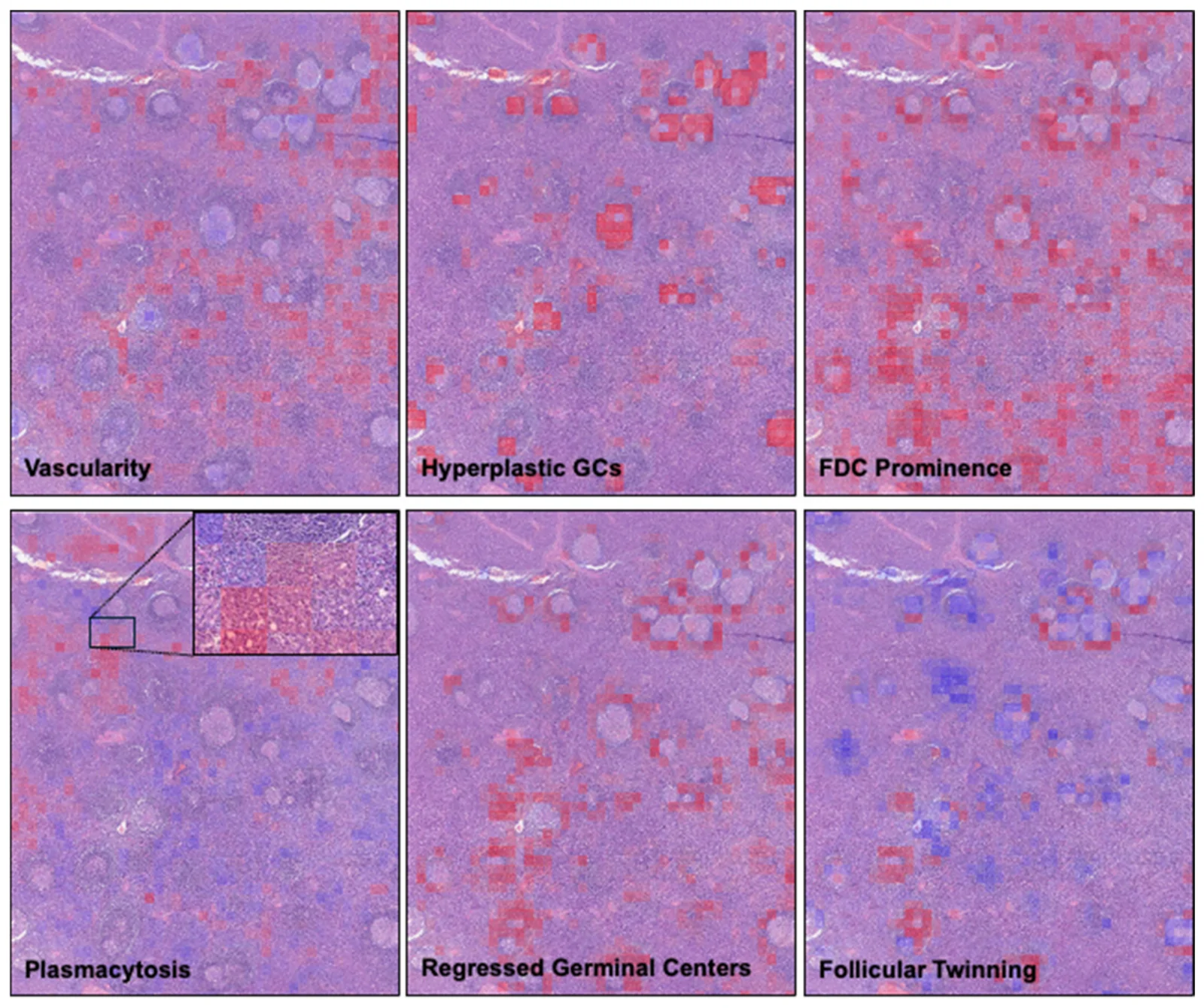
Representative feature-specific tile contribution overlays for six Castleman disease-associated histologic features. The same whole slide image is shown with separate overlays for vascularity, hyperplastic germinal centers, follicular dendritic cell prominence, plasmacytosis, regressed germinal centers, and follicular twinning, arranged from left to right and top to bottom. Red and blue tiles indicate relative signed contributions that increase or decrease, respectively, the corresponding continuous slide-level feature score. Overlay values were normalized within each feature-specific map and should not be compared quantitatively across panels. The plasmacytosis panel includes a higher magnification inset of the boxed region. These overlays provide qualitative visualizations of model weighting and were not quantitatively validated as feature localization maps, histologic segmentations, or causal explanations of the predictions.

Feature-specific tile contribution overlays were reviewed qualitatively across all six grading tasks (Figure 3). In the selected example, model weighting showed spatial correspondence with vessel-rich and plasma cell-rich interfollicular regions and with follicular regions containing the relevant architectural features. These overlays illustrate the distribution of model weighting but do not constitute quantitative localization validation, histologic segmentation, or causal explanation.

## 4. Discussion

Our study demonstrates that attention-based multiple instance learning can provide automated grading of six CD-associated histopathologic features from routine H&E whole slide images together with qualitative visualization of model weighting. Across features, the model showed moderate exact grade accuracy but higher ordinal agreement, with most disagreements limited to a single grade. This pattern is consistent with the model learning ordered morphologic signal; however, the single scanner design does not exclude slide-, site-, staining-, or scanner-associated effects.

Another central finding is the magnitude of human variability. Agreement with the consensus reference ranged from alpha = 0.20 to 0.95, and the model reference agreement of alpha = 0.56 fell within this observed range. The reference grades were established through separate grading and consensus adjudication by two hematopathologists with focused experience in CD but remain subject to uncertainty inherent in semiquantitative grading. The 0–3 categories impose discrete thresholds on morphologic findings that are continuous and spatially heterogeneous. Accordingly, model performance represents agreement with an expert consensus reference rather than absolute histopathologic truth or equivalence to a practicing hematopathologist.

The clinical motivation for reproducible histologic feature grading is clear in iMCD. Histopathology is a required component of the diagnostic evaluation, but histologic feature grades alone do not establish the diagnosis. They must be integrated with lymph node distribution, clinical and laboratory findings, virologic and ancillary studies, and exclusion of infectious, autoimmune, and neoplastic mimics. Effective therapy, nevertheless, depends on timely recognition of the syndrome and its subtype. As noted previously, in the pivotal randomized trial, durable response occurred in 34% of siltuximab-treated patients and in 0% of placebo-treated patients, illustrating the consequences of missed or delayed diagnosis. Real-world claims-based analyses demonstrate that many iMCD patients do not receive IL-6-directed therapy, which may reflect diagnostic uncertainty, delayed referral, or difficulty applying consensus criteria in non-specialist settings [2,3,11]. 

Recent retrospective studies further underscore the substantial clinical burden and heterogeneity of Castleman disease. In the BURDEN iMCD claims analysis, 140 patients with iMCD matched to 420 controls had a 6.91-fold higher prevalence of measured morbidities, with anemia, renal dysfunction, and respiratory dysfunction or interstitial lung disease among the most prevalent, and morbidity-related costs were 7.61-fold higher [10]. Although claims-based analyses are limited by coding accuracy, case-identification algorithms, and residual confounding, these findings illustrate the substantial multisystem and economic burden of iMCD. In a 20-year cohort of 217 patients, Nishimura et al. reported that median event-free survival was not reached for UCD, compared with 8.9 years for oligoCD and 2.3 years for iMCD. Among iMCD subtypes, iMCD-IPL had more favorable event-free survival than iMCD-TAFRO [29]. Together, these findings demonstrate clinically meaningful heterogeneity across Castleman disease subtypes and reinforce the importance of timely and accurate clinicopathologic assessment. The present model does not diagnose or prognosticate Castleman disease; rather, reproducible grading of individual histologic features may provide standardized inputs to the broader clinicopathologic evaluation. In a potential future decision support workflow, model-derived feature grades could populate a structured histologic assessment for pathologist confirmation or modification. Pathologist-confirmed grades could then be integrated with lymph node distribution, clinical and laboratory findings, HHV8 and other ancillary studies, and exclusion of relevant mimics. The present model does not generate a CD versus non-CD diagnosis.

From an informatics standpoint, CD poses challenges distinct from the tumor-centric tasks that dominate computational pathology. The morphologic targets are often distributed across multiple lymph node compartments, and the outputs are ordinal grades rather than binary labels. Ordinal grading is more demanding than binary classification because it requires the model to learn both the presence of a feature and its extent or severity. Our results show that the model is most reliable in a ‘near miss’ sense (high within one grade agreement), which may be the appropriate performance target when the reference standard itself is imperfect and when adjacent grades reflect subtle, continuous morphologic transitions.

Our study illustrates the practical value of foundation model embeddings for rare diseases. Large-scale weakly supervised pathology systems have historically relied on very large training cohorts, exemplified by a clinical-grade multiple-instance learning system trained on 44,732 WSIs from 15,187 patients that achieved areas under the curve (AUCs) above 0.98 for several cancer detection tasks and could potentially exclude 65–75% of slides while retaining 100% sensitivity [23]. CD is orders of magnitude rarer than these conditions, making such dataset scales unrealistic. Virchow2 partially addresses this limitation by transferring representations learned from millions of slides into a low-data setting. Other recent whole-slide foundation models, including UNI and multimodal vision-language models such as CONCH, similarly report improved data efficiency and cross-domain generalization; systematic comparisons of encoders in lymph node pathology and non-neoplastic inflammatory disorders represent an important next step [20,23,24]. 

Several feature-specific findings are instructive for future model refinement. Hyperplastic germinal centers showed the lowest exact agreement and a tendency to undercall the most severe grade. Follicular twinning similarly demonstrated limited sensitivity for grade 3. Both patterns are consistent with class imbalance and with the intrinsic difficulty of distinguishing “marked” from “moderate” involvement using a coarse ordinal rubric. In contrast, plasmacytosis showed strong ordinal fidelity with very few large errors, likely reflecting the visually distinctive nature of plasma cell-rich interfollicular zones. The relative difficulty in grading follicular twinning is not unexpected. Although hematopathologists commonly recognize follicular twinning as a feature of CD in lymph node biopsies, it is not formally incorporated into existing histologic grading frameworks, including the 2017 Blood consensus criteria or the WHO-HAEM5 classification. As a result, there is no standardized reference for assessing severity, which likely contributes to greater interobserver variability and reduced model performance for this feature. Overall, these observations suggest that subsequent iterations should incorporate imbalance-aware training strategies and compare the current regression formulation with explicitly ordinal objectives [28,30,31]. 

Model transparency is important for potential clinical use in hematopathology, where pathologists must reconcile algorithmic outputs with the underlying morphology. Tile contribution overlays may help users inspect where model weighting is concentrated, but they are not equivalent to causal explanations or validated feature localization and may reflect relevant morphology, tissue composition, staining or scanner effects, or other confounders. We therefore regard them as qualitative and hypothesis-generating. Prospective studies should evaluate whether access to model grades and overlays improves pathologist efficiency, consistency, or confidence in realistic workflows [22,25]. 

Clinical implementation would require local validation across scanner and staining conditions, appropriate tissue and image quality control, prospective workflow evaluation, and ongoing monitoring for performance drift and subgroup performance. The model should initially function as pathologist-facing decision support, with pathologist review and override and defined procedures for discordant outputs, quality control failures, escalation, and accountability. Because access to whole slide imaging infrastructure and subspecialty hematopathology expertise is uneven, implementation studies should assess whether the technology reduces or widens disparities in access and performance [25].

We note that our study has limitations. Although tissue originated from multiple institutions, all slides were digitized using a single scanner, and tissue processing and H&E staining followed routine local protocols that were not prospectively harmonized. Performance in unseen scanner, staining, and preanalytic domains, therefore, remains unknown. The cohort remains modest in size by deep learning standards and may not capture the full range of patient populations and specimen quality encountered in practice. The expert consensus reference was established through separate grading and adjudication but remains subject to uncertainty inherent in semiquantitative grading of continuous, spatially heterogeneous morphology. We focused on grading individual morphologic features rather than diagnosing CD, assigning subtypes, or separating CD from specific mimics. The available cohort is not suitable for estimating diagnostic accuracy because the CD-associated group includes both CD and CD-like histology, whereas the comparison group was defined by the absence of a pathology diagnosis or diagnostic comment identifying or suggesting CD and the absence of clinical suspicion, rather than by systematic diagnostic adjudication against relevant mimics. Evaluation of diagnostic utility will require external, cross-scanner, and prospective studies with rigorously adjudicated labels, appropriate mimics, and integration of pathologist-confirmed feature grades with clinical, laboratory, imaging, virologic, immunophenotypic, and exclusion criteria [4,26]. 

In summary, an attention-based MIL model leveraging a large pathology foundation model can generate reproducible ordinal grades of CD-associated histopathologic features on routine H&E slides, together with qualitative visualization of model weighting. This capability could support standardization in multicenter studies and provide reproducible histologic inputs for future diagnostic workflows, including settings without deep subspecialty expertise. The current model should not be interpreted as a standalone diagnostic system. Future work will emphasize prospective validation, encoder comparisons, uncertainty calibration, and evaluation of whether model-derived feature grades improve clinicopathologic assessment when combined with adjudicated clinical, laboratory, imaging, and ancillary pathology data.

## Figures and Tables

**Table 1:** Reference grade distributions in the held-out evaluation set (*n* = 142 WSIs).

Histologic Feature	Grade 0, *n* (%)	Grade 1, *n* (%)	Grade 2, *n* (%)	Grade 3, *n* (%)
Regressed germinal centers	21 (14.8)	21 (14.8)	40 (28.2)	60 (42.3)
Follicular dendritic cell prominence	33 (23.2)	52 (36.6)	34 (23.9)	23 (16.2)
Vascularity	18 (12.7)	48 (33.8)	45 (31.7)	31 (21.8)
Hyperplastic germinal centers	84 (59.2)	27 (19.0)	21 (14.8)	10 (7.0)
Plasmacytosis	27 (19.0)	76 (53.5)	26 (18.3)	13 (9.2)
Follicular twinning	86 (60.6)	20 (14.1)	18 (12.7)	18 (12.7)

**Table 2:** Pairwise concordance with reference graders (Krippendorff’s alpha).

Rater	Krippendorff’s Alpha
Pathologist 1	0.419
Pathologist 2	0.952
Pathologist 3	0.523
Pathologist 4	0.431
Pathologist 5	0.524
Pathologist 6	0.449
Pathologist 7	0.203
Model	0.560

## Data Availability

De-identified data supporting the findings of this study, including derived tile embeddings and slide-level feature grades, may be made available by the corresponding author upon reasonable request and subject to institutional and regulatory approvals.

## Abbreviations

CD, Castleman disease; FDC, follicular dendritic cell; H&E, hematoxylin and eosin; HHV-8, human herpesvirus 8; IL-6, interleukin 6; iMCD, idiopathic multicentric Castleman disease; MIL, multiple-instance learning; POEMS, polyneuropathy, organomegaly, endocrinopathy, monoclonal plasma cell disorder, and skin changes; UCD, unicentric Castleman disease; HSV, hue, saturation, value; WSI, whole slide image.
